# Enhanced recovery after surgery for cesarean delivery: a systematic review and meta-analysis of randomized trials with trial sequential analysis

**DOI:** 10.1016/j.bjane.2026.844817

**Published:** 2026-08-14

**Authors:** Tauãna Terra Cordeiro de Oliveira, Vanessa Tapioca, Eduardo Pereira, Bruna Ferreira, Tamiris Soares, Sara Amaral, Maria Luisa Assis, Gabriel Carvalho, Julio S. Curi, Kamal Kumar

**Affiliations:** a Department of Medicine, Universidade Federal de São João del-Rei, Divinópolis, MG, Brazil; b Department of Anesthesiology, Duke University Medical Center, Durham, USA; c Department of Medicine, Universidade Federal de Minas Gerais, Belo Horizonte, MG, Brazil; d Department of Anesthesiology, Universidade da Região de Joinville, Joinville, SC, Brazil; e Department of Anesthesiology and Perioperative Medicine, University of Western, Ontario, Canada; f Orthomed Anesthesia, Addison, Texas, USA; g Department of Anesthesiology, Boston Medical Center, Boston, USA; h Department of Anesthesiology, Hospital Cariacica Meridional, Cariacica, ES, Brazil; i Department of Medicine, Universidade do Vale do Taquari, Lajeado, RS, Brazil; j Department of Anesthesia & Perioperative Medicine, Schulich School of Medicine & Dentistry, Western University, Ontario, Canada

**Keywords:** Cesarean section, Enhanced recovery after surgery, Length of stay, Maternal health, Perioperative care, Postoperative care

## Abstract

**Background:**

Enhanced Recovery After Surgery (ERAS) protocols have improved perioperative care across multiple surgical specialties. In obstetrics, ERAS pathways for cesarean delivery have been increasingly adopted; however, their comparative effectiveness versus standard care remains uncertain. Therefore, we conducted a systematic review and meta-analysis with trial sequential analysis to compare ERAS protocols with standard perioperative care in cesarean delivery.

**Methods:**

We systematically searched MEDLINE, Embase, Web of Science, and Cochrane databases for Randomized Clinical Trials (RCTs) comparing ERAS and standard care in parturient undergoing cesarean delivery. Statistical analyses were performed using R software (version 4.2.2). We conducted subgroup analyses based on cesarean category (elective vs. emergency) and national income level. A Trial Sequential Analysis (TSA) was also performed.

**Results:**

We included 13 RCTs comprising 1,809 parturient. ERAS was associated with a significant reduction in hospital Length of Stay (LOS) compared with standard care (MD = -17.05-hours, 95% CI -25.08 to -9.02 hours, *I*^2^ = 98.2%, n = 1,128, p < 0.01), with similar effects across elective and emergency cesarean deliveries. Greater reductions were observed in low- and middle-income countries, whereas no significant difference was identified in upper-middle-income settings, albeit formal subgroup test was not significant. ERAS was associated with lower postoperative pain at 24- and 48 hours, reduced wound infection, and higher maternal satisfaction, with no differences in readmission and postoperative nausea and vomiting. TSA suggested that the accrued sample size was adequate for hospital LOS; however, this should be interpreted cautiously given the substantial clinical and methodological heterogeneity.

**Conclusion:**

This systematic review and meta-analysis suggest that ERAS protocols may be associated with reduced hospital stay and improved maternal recovery after cesarean delivery.

## Introduction

Surgical care is a fundamental component of global health, as almost one-third of the global disease burden requires surgical intervention.^1^ The demand for surgical procedures, particularly cesarean deliveries, has risen substantially worldwide. By 2030, cesarean deliveries are projected to account for about 30% of all births, totaling approximately 38 million procedures. Most of these procedures are expected to occur in Low- and Middle-Income Countries (LMIC), compared with 11.7% in high-income countries.^2^ This trend raises public health concerns, as cesarean delivery outcomes are a key indicator of maternal health system performance.

Enhanced Recovery After Surgery (ERAS) protocols are perioperative care pathways designed to improve patient experience and surgical care. First introduced in 2001, ERAS has become widely adopted across multiple surgical specialties and has consistently demonstrated benefits in safety and clinical outcomes.^3^ In obstetrics, Enhanced Recovery After Cesarean (ERAC) pathways were introduced in 2013,^4^ and updated evidence-based perioperative care recommendations for cesarean delivery were published by the ERAS Society in 2025.^4^, ^5^, ^6^ Standardization of these pathways has been associated with improvements in quality of care, patient safety, and cost-effectiveness in healthcare systems.^1^ Therefore, ERAS may represent a potential strategy to strengthen surgical systems in resource-limited settings without requiring substantial additional resources. Although high-quality evidence remains limited, several ERAS components appear to be low risk, demonstrate favorable clinical outcomes, and align with maternal preferences, particularly due to their association with shorter hospital stays.^7^

Previous meta-analyses have assessed the impact of ERAS implementation on cesarean delivery.^8^, ^9^, ^10^, ^11^ However, their conclusions were limited by the inclusion of observational studies, the absence of stratification by country income level and cesarean type, and the lack of Trial Sequential Analysis (TSA) and Minimal Clinically Important Difference (MCID) assessments. Therefore, we conducted an updated systematic review and meta-analysis of Randomized Controlled Trials (RCTs) with TSA to compare ERAS protocols with standard perioperative care in patients undergoing cesarean delivery.

## Methods

This study was conducted and reported following the Preferred Reporting Items for Systematic Reviews and Meta-analyses (PRISMA) statement and the Cochrane Handbook for Systematic Reviews of Interventions recommendations.^12^^,^^13^ The protocol was prospectively registered in the International Prospective Register of Systematic Reviews (PROSPERO) under identifier CRD420251152114 on October 23, 2025.

### Eligibility criteria

We included studies that met the following eligibility criteria: 1) Were RCTs, 2) Compared ERAS protocols with standard care, and 3) Included patients aged 18 years or older undergoing cesarean delivery. We excluded studies with overlapping populations, studies not written in English, and studies assessing modified or isolated pharmacological or nutritional interventions that did not constitute a comprehensive ERAS protocol.

### Study selection and data extraction

We systematically searched the MEDLINE, Cochrane, Embase, and Web of Science databases on November 17, 2025. A detailed search strategy is available in Supplementary Table S1. Study selection was performed by two independent reviewers using the Rayyan software (rayyan.ai), with disagreements resolved by consensus with a third author. The reference lists of included articles were manually screened to identify additional studies (“backward snowballing” technique). After selection, data were recorded using a piloted, standardized extraction form. Two authors independently extracted quantitative variables, and three authors extracted qualitative data. Data were extracted for the following variables: author and year of publication; country; sample size in the ERAS and standard care groups; gestational age; type of cesarean section (elective, emergency, or mixed); definitions of outcomes; and details of the ERAS protocol implemented across the preoperative, intraoperative, and postoperative phases. For each RCT, information on primary and secondary outcomes was retrieved, along with measures of variance, timing of assessment, and scale type. Discrepancies were resolved by another author when necessary. If clarification was required, the corresponding authors of the original studies were contacted by email.

### Quality and risk of bias assessment

The Cochrane Risk of Bias 2 (RoB 2) tool^14^ was utilized to assess the studies’ risk of bias based on the five domains: 1) Randomization process, 2) Deviations from intended intervention, 3) Missing outcome data, 4) Measurement of the outcome, and 5) Selection of the reported result. Risk of bias figures were generated using the RobVis visualization tool.^15^

The Grading of Recommendations Assessment, Development and Evaluation (GRADE) approach was used to assess the certainty of the evidence and the level of recommendation for each outcome in the meta-analysis.^16^ This tool accounts for several domains, including risk of bias, imprecision, inconsistency (heterogeneity), indirectness, and publication bias. Any disagreements regarding the certainty of evidence and risk of bias assessments were resolved by a third author.

### Endpoints and subgroup analysis

The primary endpoint was hospital Length Of Stay (LOS), defined as the duration of postoperative hospitalization until discharge or fulfillment of institutional discharge criteria. Secondary endpoints were postoperative pain scores at 24 and 48 hours (at rest), Postoperative Nausea and Vomiting (PONV), readmission rate, wound (surgical site) infection, and overall maternal satisfaction. Definitions for all endpoints are provided in Supplementary Table S2.

Maternal satisfaction was assessed using standardized tools, including a five-point Likert scale, the Patient Satisfaction with Nursing Care Quality Questionnaire (PSNCQQ), and the Visual Analogue Scale (VAS). Additionally, studies that used non-standardized methods based on a 0−10 numeric rating were also included. For quantitative synthesis, all satisfaction scores were rescaled to a common 0−10 scale; higher scores indicated greater satisfaction. Postoperative pain scores were also standardized using the 0−10 VAS (0 = no pain; 10 = worst pain).

We conducted two prespecified subgroup analyses. Trials were stratified by national income level as LMIC versus Upper-Middle-Income Countries (UMIC) based on the World Bank classification for the trial’s country.^17^ Studies were also stratified by cesarean category as emergency, elective, or mixed cohorts.

### Data synthesis and analysis

Continuous outcomes were synthesized using the inverse variance method with a random-effects model and are reported as Mean Differences (MD) or Standardized Mean Differences (SMD) with 95% Confidence Intervals (95% CI). Dichotomous outcomes were pooled using the Mantel-Haenszel method with a random-effects model and are presented as Risk Ratios (RR) with 95% CI. Forest plots were generated to illustrate treatment effects. Outcomes reported by at least three trials including more than 100 participants were quantitatively synthesized, whereas the remaining outcomes were described qualitatively. All statistical tests were two-sided, with p < 0.05 considered statistically significant. When studies reported medians with interquartile ranges or minimum and maximum values, data were converted following Wan's method.^18^

Heterogeneity was assessed using Cochran’s *Q*-test and the *I*^2^ statistic, with the DerSimonian-Laird method. The degree of heterogeneity was interpreted using the Cochrane thresholds: low (0 to 40%), moderate (30% to 60%), substantial (50% to 90%), and considerable (75% to 100%).^13^ All statistical analyses were performed under a random-effects model using the R software (packages *meta, metafor*, and *dmetar*).^19^^,^^20^

We performed TSA for the primary endpoint to assess the risks of type I and type II errors and estimate the required sample size for the outcome. We set the thresholds for the *Z*-score with the O’Brien-Fleming alpha spending function and applied a random-effects model using the DerSimonian-Laird method, which allowed a type I error of 0.05 and a type II error of 0.20 (power = 80%). All calculations were performed using software TSA 0.9.^21^

To explore inconsistencies among studies, we also performed a sensitivity analysis by omitting each study individually for each endpoint (leave-one-out technique) using a random-effects model.

### Interpretation of results

Pain scores were interpreted using a broader perspective, as our analysis included emergency cesarean sections. As there is no MCID specific to post‑cesarean pain assessment, we referred to evidence from gynecologic surgery, specifically hysterectomy, as the closest comparable context. In this context, we considered evidence suggesting that a change of approximately 1.5 on the VAS typically reflects the MCID in acute postoperative pain.^22^

For maternal satisfaction, a 0.7−1.1-point difference on a 0−10 patient VAS, equivalent to a 7−11 mm change on the original 100-mm scale, was considered the MCID threshold for a perceptible improvement in satisfaction.^23^

## Results

### Study selection and characteristics

**Figure 1:**
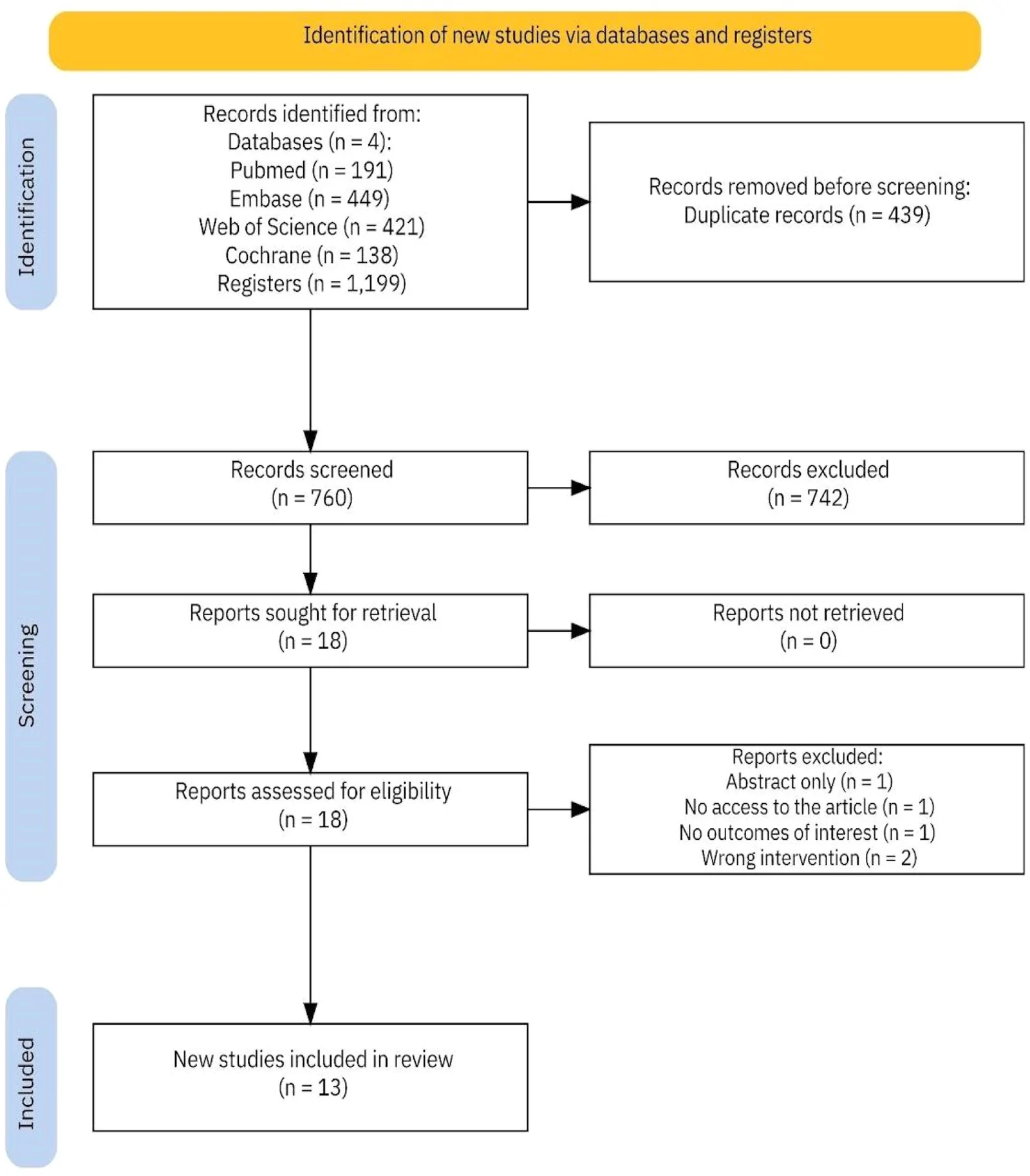
PRISMA flow diagram of study screening and selection.

Our search strategy identified 1,199 studies, of which 13 trials including 1,809 parturient ultimately met the inclusion criteria.^24^, ^25^, ^26^, ^27^, ^28^, ^29^, ^30^, ^31^, ^32^, ^33^, ^34^, ^35^, ^36^ The full details of the study selection process are shown in Figure 1. A list of excluded studies and reasons for exclusion is available in Supplementary Table S3. Three trials were rated low risk of bias^25^, ^27^, ^28^ while the remaining ten were assessed as having some concerns.^24^^,^^26^^,^^29^, ^30^, ^31^, ^32^, ^33^, ^34^, ^35^, ^36^ Overall, the included trials were judged to present a moderate risk of bias (Supplementary Fig. S1).

Among the 13 included RCTs, 908 parturient (50.19%) were allocated to the ERAS group. Nine studies enrolled parturient undergoing elective cesarean delivery (n = 1,207),^26^, ^27^, ^28^, ^29^, ^30^, ^31^^,^^33^, ^34^, ^35^ three enrolled parturient undergoing emergency cesarean delivery (n = 402),^24^^,^^25^^,^^32^ and one included both elective and emergency cases (n = 200).^36^ Eight trials (61.54%) were conducted in LMIC.^24^^,^^25^^,^^27^, ^28^, ^29^^,^^31^^,^^32^^,^^34^ ERAS protocols varied across studies, and details of the intervention and study characteristics are summarized in Table 1.

**Table 1:** Baseline characteristics of the included studies.

Author, year	Country	Sample size (n) ERAS vs. Standard Care	Gestational age [mean (SD)]	Type of cesarean	ERAS protocol		
					Preoperative	Intraoperative	Postoperative
Abilashini, 2025	India	50 vs. 50	36.2 (1.44)	Emergency	Education; hydration optimization; early mobilization.	Multimodal analgesia; minimally invasive techniques; normothermia.	Oral intake and mobilization within 6h; emphasis on non‐opioid analgesia for pain.
Baluku, 2020	Uganda	80 vs. 80	NA	Emergency	Education; no fasting (solids and liquids); antibiotic prophylaxis; prophylaxis against postoperative NV and pulmonary aspiration.	Spinal hyperbaric bupivacaine and 100 μg of ITM; restrictive fluid administration; continuous adrenaline infusion; normothermia (warm IV fluids); local wound infiltration; rectal diclofenac and misoprostol.	Carbohydrate drink and cessation of IV fluids within 1h; early breastfeeding; oral single fixed-dose (ibuprofen, acetaminophen); IV pethidine (breakthrough pain); early mobilization; early urethral catheter removal; oral antibiotics.
Birden, 2025	Turkey	78 vs. 78	38.33 (1.41)	Elective	Clear liquids until 2h before surgery; light meal up to 6h before surgery; oral carbohydrate supplementation 2h before CD; IV antibiotics within 1h before the skin incision.	Warming devices (hypothermia); euvolemia is prioritized; fluid preloading, IV phenylephrine or ephedrine, and lower limb compression to reduce hypotension and perioperative NV; multimodal antiemetic agents.	Chewing gum after CD; multimodal analgesia (regular nonsteroidal anti-inflammatory drugs and paracetamol); multimodal bowel regimen; regular diet within 2h after CD; tight control of capillary blood glucose; pneumatic compression stockings (prevent thromboembolic disease); early mobilization; urinary catheters removed immediately after CD.
Darwish, 2022	Egypt	150 vs. 150	NA	Elective	Preparatory phase of detailed explanation of the objective of ERAS; breastfeeding education; stop solid food at least 6‒8h and clear oral fluid at least 2h before CD; prophylactic antibiotics; thromboprophylaxis measures.	Proper fluid balance: active warming (preheated IV fluids and cotton blankets); gum chewing; delayed cord clamping; NV prophylaxis; immediate skin-to-skin contact/ breastfeeding.	Continuous gum chewing; NV prophylaxis; early oral intake; parenteral analgesics regularly; early mobilization; removal of Foley’s catheter 2h after CD; early removal of dressing; meticulous monitoring of vital signs; lactation consultation interview; community support (midwives visit; physiotherapists) and opportunity to go home on day one.
Emelinda, 2024	Cameroon	21 vs. 21	NA	Elective	Information on the procedure, pain management plan, necessity of early feeding and mobilization, lactation, and discharge criteria; sweetened fluids up to 2h and solid foods up to 6h before surgery; standard regional anesthetic technique (spinal anesthesia).	Cefuroxime 60 min before incision; Dexamethasone 8 mg + 8 mg Ondansetron for NV; 50 mg Ranitidine; pre‐load of fluids + intermittent ephedrine boluses; covering of patient’s chest and crystalloid warming (to 37°).	Balanced analgesia with NSAIDs and Paracetamol; Chewing of gum; regular feeding within 2h from surgery; compression stockings and heparin for thromboprophylaxis; early mobilization; sitting 2h after surgery, and walking 6h after removal of urinary catheter; removal of the urinary catheter 6h after CD.
Kanabar, 2024	India	46 vs. 46	39.64 (1.08)	Elective	ERAS education; oral glucose-rich clear fluid 2h before CD; prophylactic antibiotics; 8 mg of IV dexamethasone, 4 mg IV ondansetron for NV prophylaxis; prophylaxis against pulmonary aspiration (40 mg of IV pantoprazole and 10 mg of IV metoclopramide).	Single-shot spinal anesthesia with hyperbaric bupivacaine and 15 μg of preservative-free fentanyl; restrictive fluid to ensure normovolemia; 6 mg intravenous boluses of mephentermine (hypotension); warm IV fluids and warm clothing cover.	Bilateral TAP block; diclofenac 100 mg (rectal); oral carbohydrate drink and cessation of IV fluids within 1h; early breastfeeding (30-min); fixed-dose 400 mg of ibuprofen and 500 mg of acetaminophen every 8h, IV tramadol (breakthrough pain); early mobilization at 8h; Early urethral catheter removal at 6h; oral antibiotics.
Klangprapan, 2022	Thailand	22 vs. 21	38.7 (0.5)	Elective	Education; 50 mL of water with 50 g of glucose at 6 a.m. on the day of surgery.	Standardized spinal anesthesia; cefazolin prophylaxis (both groups); metoclopramide 10 mg IV after spinal; euvolemia and hypothermia prevention.	Oral fluids/food ≥ 2h once stable; scheduled multimodal analgesia; early ambulation; catheter out at 6h.
Kulshrestha, 2025	India	70 vs. 70	37.4 (2.07)	Elective	Counseling in 3rd trimester and on admission; fasting 6h solids, clear fluids 2–3h pre-op (carb-rich allowed); prophylactic antibiotics; aspiration prophylaxis (ranitidine + metoclopramide).	Standardized spinal anesthesia; TAP block after closure; crystalloids co-load; prophylactic phenylephrine infusion; chlorhexidine-alcohol skin prep; normothermia; immediate skin-to-skin; delayed cord clamping; PONV prophylaxis.	Scheduled multimodal analgesia; ondansetron prophylaxis; oral fluids 2h, regular diet same day; gum chewing; mobilization on the first day; breastfeeding < 1h; catheter removal < 12h; IV fluids stopped in ward.
Mundhra, 2024	India	71 vs. 71	37.91 (1.90)	Emergency	NA	Standardized spinal anesthesia; goal-directed fluids; normothermia.	Oral fluids at 2h, normal diet at 8h, gum chewing; early mobilization, catheter removal 6–12h; ceftriaxone + cefixime, metronidazole; multimodal analgesia; incentive spirometry; dressing off at 24h.
Pan, 2020	China	112 vs. 104	38.99 (0.90)	Elective	Education and counseling, reduced fasting (6h solids, 2h fluids), antibiotics, aspiration prophylaxis.	CSEA; prophylactic phenylephrine 50 µg; active warming with warm-air blower; oxytocin infusion; carboprost if needed.	Early oral intake (2h), early mobilization (6h turning, ambulation on the first day), skin-to-skin, breastfeeding < 1h, scheduled PCEA with 6 mg morphine (vs. 6–7 mg in controls), multimodal antiemetic prophylaxis, ondansetron/tropisetron.
Rajendran, 2025	India	50 vs. 50	NA	Elective	Patient education on pain, feeding, mobilization, lactation, discharge, and follow-up. Clear fluids up to 2h before surgery.	Spinal anesthesia with 0.5% bupivacaine heavy (10 mg) + buprenorphine (60 μg). Prophylactic phenylephrine (100 μg) to prevent hypotension. Granisetron for PONV prophylaxis. Normothermia actively maintained (forced-air warming, pre-warmed bed, fluid warmer). Standard uterotonics and antibiotics.	TAP block with ropivacaine 0.5% (20 mL each side) + dexamethasone (4 mg). Oral paracetamol; tramadol (breakthrough pain). Oral intake within 2h, early mobilization at 6h, immediate skin-to-skin, breastfeeding within 1h.
Teigen, 2020	USA	58 vs. 60	39.13 (0.27)	Elective	Standard institutional prophylaxis (antibiotics, surgical preparation). All patients received neuraxial anesthesia (spinal, epidural, or CSEA).	Standard cesarean technique per attending surgeon. No difference in anesthesia between groups.	Chewing gum (xylitol) to reduce ileus; scheduled IV ketorolac for 24h; early oral intake (clear liquids in PACU, regular diet within 30-min); early urinary catheter removal at 12h; dressing removal at 6h; early mobilization at 12h; incentive spirometry every 8h.
Xu, 2025	China	100 vs. 100	NA	Mixed	Reduced fasting (6h solids, 2h clear liquids); oral ranitidine, antacids, acetaminophen; prophylactic antibiotics (IV cefazolin ± oral azithromycin); alcohol-based chlorhexidine/iodine; group education on diet, exercise, and glucose monitoring.	Neuraxial anesthesia; local wound infiltration; goal-directed fluid therapy; active warming (blankets + warmed IV fluids); IV insulin if glucose > 8.25 mmoL.L^-1^; 10% glucose infusion.	Early oral intake (sips of water within 2h, full diet within 8h); chewing gum every 8h; early mobilization (sitting within 8h, ambulation by 24h); urinary catheter removal at 8–12h; early physiotherapy; multimodal analgesia including intermittent IV morphine.

a.m., Ante meridiem; CD, Cesarean Delivery; CSEA, Combined Spinal and Epidural Anesthesia; ERAS, Enhanced Recovery After Surgery; g, Grams; h, Hour(s); ITM, Intrathecal Morphine; IV, Intravenous; mg, Milligrams; μg, Micrograms; mL, Milliliters; min: minutes; mmoL.L^-1^, Millimoles per Liter; NA, Not Available; NSAIDs, Nonsteroidal Anti-Inflammatory Drugs; NV, Nausea and Vomiting; PACU, Post-Anesthesia Care Unit; PCEA, Patient-Controlled Epidural Analgesia; PONV, Postoperative Nausea and Vomiting; SD, Standard Deviation; TAP, Transversus Abdominis Plane.

### Main endpoint – Hospital length of stay

**Figure 2:**
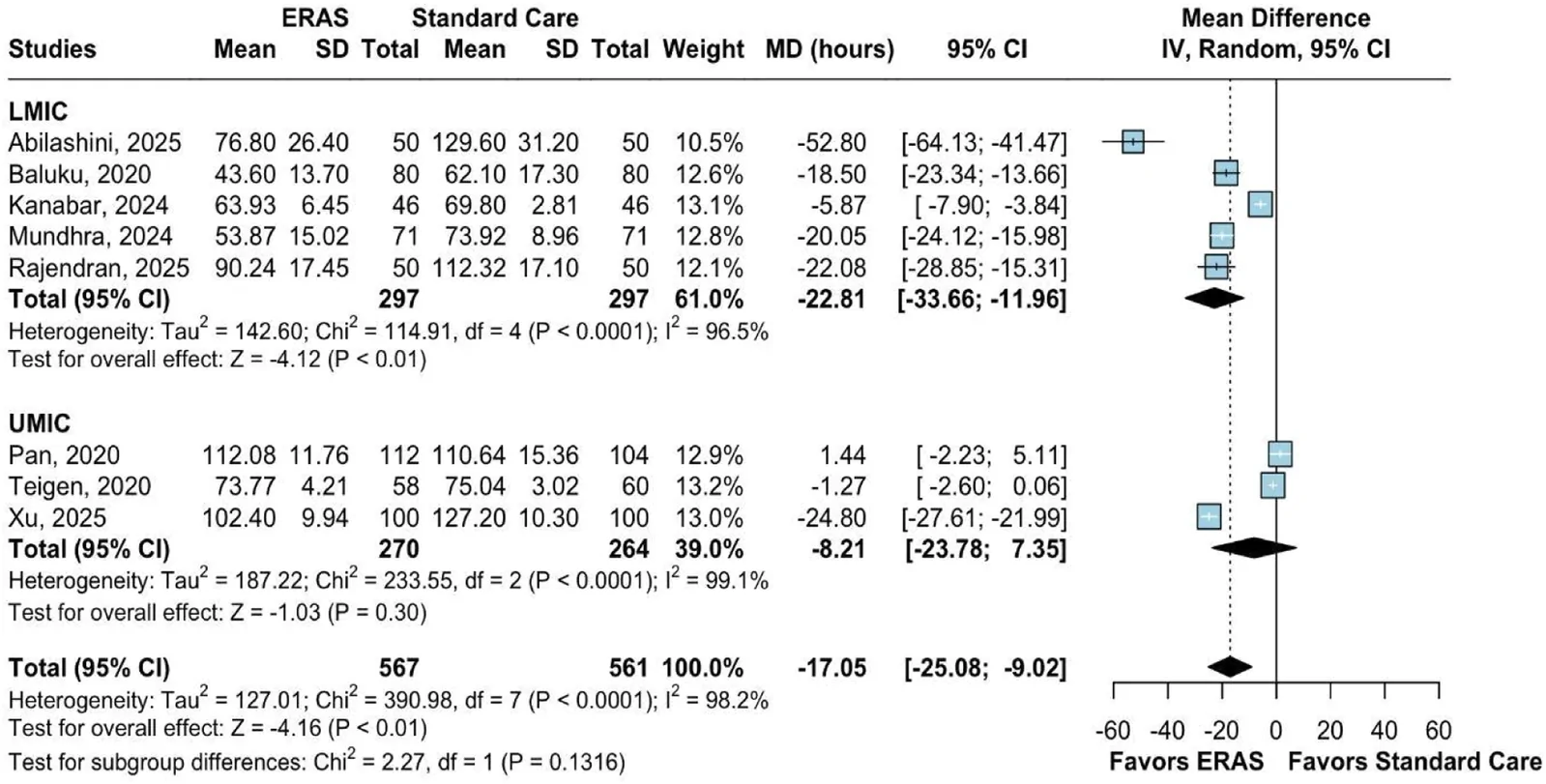
Forest plot depicting the endpoint of hospital length of stay, stratified by country income level. The plot displays mean differences with 95% Confidence Intervals for each included study and subgroup-specific pooled random-effects estimates. Abbreviations: ERAS, Enhanced Recovery After Surgery; SD, Standard Deviation; CI, Confidence Interval; MD, Mean Difference; LMIC, Low- and Middle-Income Countries; UMIC, Upper-Middle-Income Countries.

Eight RCTs, including 1,128 participants, reported hospital LOS.^24^^,^^25^^,^^29^^,^^32^, ^33^, ^34^, ^35^, ^36^ ERAS protocols significantly shortened LOS compared with standard care (MD = -17.05 hours, 95% CI -25.08 to -9.02 hours, *I*^2^ = 98.2%, p < 0.01, Fig. 2). Due to the high heterogeneity observed in the pooled analysis, a leave-one-out sensitivity analysis showed that the pooled effect remained stable after sequential exclusion of each study (Supplementary Fig. S2). We rated the overall GRADE certainty of the evidence as very low, primarily because of inconsistency and risk of bias (Supplementary Table S4).

Subgroup analysis by cesarean type indicated a reduction in hospital LOS across all categories (emergency: MD = -29.04 hours, 95% CI -42.34 to -15.74, *I*^2^ = 93.6%, p < 0.01; elective: MD = -5.9-hours, 95% CI -11.2 to -0.6 hours, *I*^2^ = 94%, p = 0.03; Supplementary Fig. S3), with significant differences between subgroups (p < 0.01). When stratified by country income level, ERAS significantly reduced hospital LOS in LMIC (MD = -22.81 hours, 95% CI -33.66 to -11.96 hours, *I*^2^ = 96.5%, p < 0.01, Fig. 2).^24^^,^^25^^,^^29^^,^^32^^,^^34^ In contrast, no statistically significant difference was observed in UMIC (MD = -8.21 hours, 95% CI -23.78 to 7.35 hours, *I*^2^ = 99.1%, p = 0.3; Fig. 2),^33^^,^^35^^,^^36^ although the test for subgroup differences did not achieve significance to show substantial variability between groups (p = 0.13).

**Figure 3:**
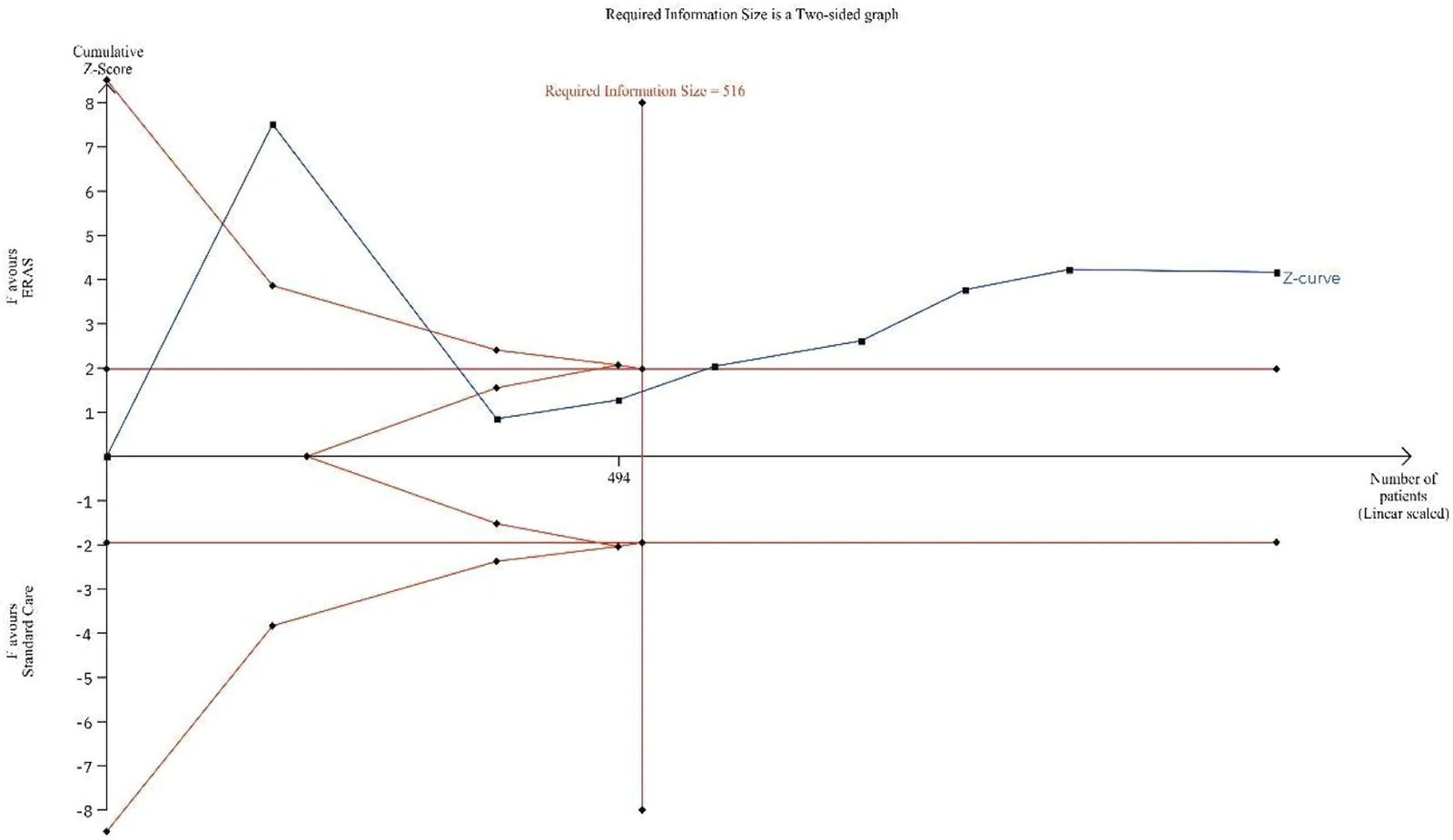
Trial sequential analysis for the hospital length of stay. ERAS, Enhanced Recovery After Surgery.

TSA for hospital LOS showed that the cumulative *Z*-curve crossed the monitoring boundaries, indicating low risks of type I and type II errors (Fig. 3) and suggesting that the pooled sample size was sufficient to draw reliable conclusions for this outcome.

### Secondary endpoints

#### Pain score

**Figure 4:**
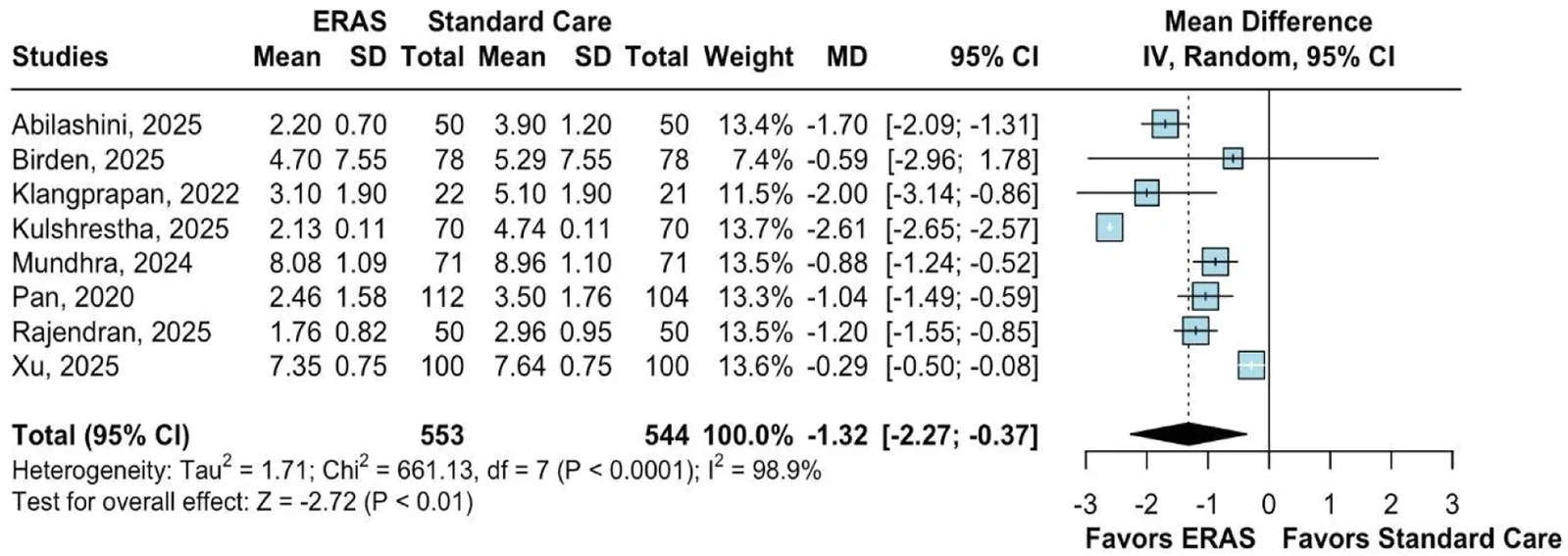
Forest plot for the outcome of pain scores at rest at 24 hours. The plot displays mean differences with 95% Confidence Intervals for each included study and the pooled random-effects estimate. ERAS, Enhanced Recovery After Surgery; SD, Standard Deviation; CI, Confidence Interval; MD, Mean Difference.

**Figure 5:**
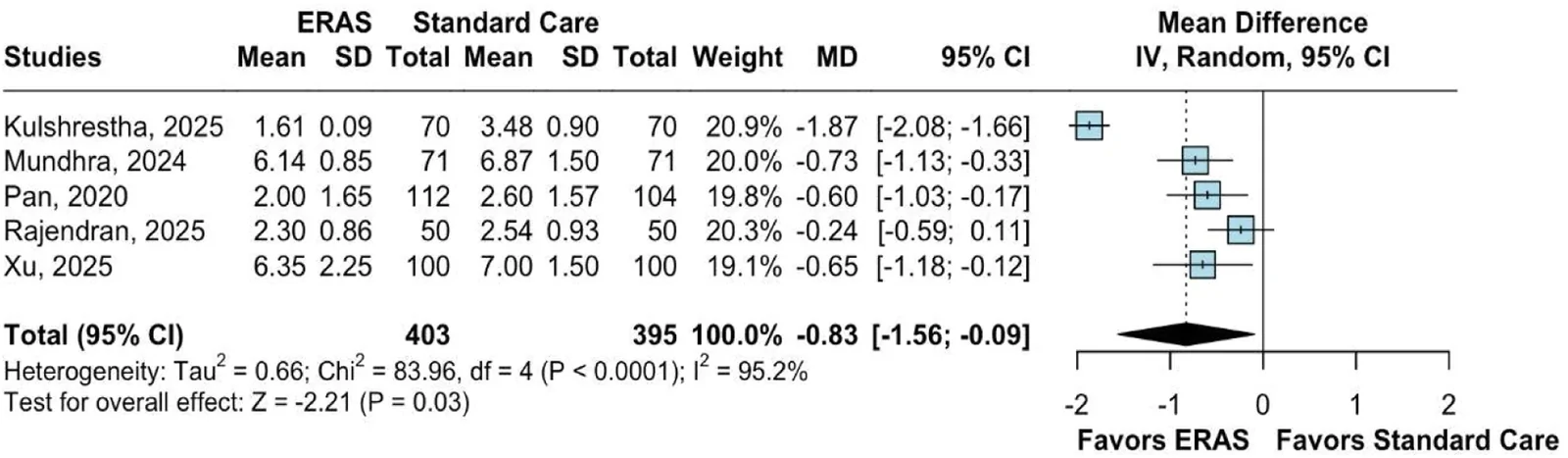
Forest plot depicting the endpoint of pain scores at rest at 48 hours. The plot displays mean differences with 95% Confidence Intervals for each included study and the pooled random-effects estimate. ERAS, Enhanced Recovery After Surgery; SD, Standard Deviation; CI, Confidence Interval; MD, Mean Difference.

Analysis of pain scores showed lower scores with ERAS protocol at rest at both 24 hours (MD = -1.32 points, 95% CI -2.27 to -0.37, *I*^2^ = 98.9%, p < 0.01, n = 1,097; Fig. 4)^24^, ^26^, ^30^, ^31^, ^32^, ^33^, ^34^, ^36^ and 48 hours (MD = -0.83-points, 95% CI -1.56 to -0.09, *I*^2^ = 95.2%, p = 0.03, n = 798; Fig. 5).^31^, ^32^, ^33^, ^34^^,^^36^ The GRADE certainty of evidence for both time points was rated as very low due to risk of bias, inconsistency, and imprecision (Supplementary Table S4). Using an MCID of about 1.5 VAS points,^22^ the reductions at 24 hours and 48 hours did not meet the threshold for clinical relevance. The leave-one-out sensitivity analysis at each time point demonstrated that the direction and significance of the effect remained consistent across trials, even after the exclusion of the two studies that incorporated a transversus abdominis plane block (Supplementary Fig. S2).^31^^,^^34^

#### Wound infection

Pooled data from four RCTs showed that ERAS significantly reduced wound infections compared with standard care (RR = 0.23, 95% CI 0.07 to 0.73, p = 0.01, *I*^2^ = 0%, n = 727; Supplementary Fig. S4).^25^, ^26^, ^27^^,^^35^ The result remained consistent in the leave-one-out sensitivity analysis (Supplementary Fig. S2). The GRADE certainty of evidence was rated as moderate, downgraded by one level due to the small number of studies contributing to this outcome (Supplementary Table S4).

#### Readmission

Five RCTs assessed readmission.^25^^,^^26^^,^^28^^,^^32^^,^^35^ A pooled analysis found no statistically significant difference between ERAS protocols and standard care groups (RR = 0.59, 95% CI 0.23 to 1.55, p = 0.29, *I*^2^ = 13.2%, n = 611; Supplementary Fig. S5). Sensitivity analyses yielded similar results (Supplementary Fig. S2). The GRADE certainty of evidence was rated very low, downgraded for risk of bias, imprecision, and publication bias (Supplementary Table S4).

#### PONV

Pooled analysis of five RCTs showed that ERAS protocols were not associated with a significant reduction in PONV compared with standard care (RR = 0.65, 95% CI 0.36 to 1.19, *I*^2^ = 0%, p = 0.16, n = 667; Supplementary Fig. S6).^25^^,^^26^^,^^28^^,^^33^^,^^34^ Sensitivity analyses demonstrated a consistent direction of effect (Supplementary Fig. S2), and the GRADE certainty of the evidence was rated as very low due to risk of bias, imprecision, and potential publication bias (Supplementary Table S4).

#### Maternal satisfaction score

The pooled analysis of five studies demonstrated a statistically significant improvement favoring ERAS protocols (SMD = 1.02, 95% CI 0.33 to 1.72, *I*^2^ = 94.9%, p < 0.01, n = 756; Supplementary Fig. S7).^24^^,^^31^^,^^33^^,^^34^^,^^36^ Leave-one-out sensitivity analysis demonstrated the stability of the pooled effect (Supplementary Fig. S2). The observed improvement was clinically meaningful, as point estimates consistently exceeded the MCID of approximately 0.7−1.1 points on a 0−10 VAS, corresponding to a 7−11 mm change on the original 100 mm scale.^23^ However, due to substantial heterogeneity, concerns about risk of bias, and the limited number of trials reporting this outcome, the GRADE certainty of the evidence was rated as very low (Supplementary Table S4).

## Discussion

In this systematic review and meta-analysis of 13 RCTs including 1,809 parturients and comparing ERAS protocols with standard perioperative care for cesarean delivery, we found that ERAS protocols were associated with a statistically significant reduction in hospital LOS, lower postoperative pain scores at rest at both 24 and 48 hours, lower incidence of wound infection, and higher maternal satisfaction. No significant differences in readmission rates or PONV were found.

ERAS protocols were introduced in 1997 by Henrik Kehlet, and over the years, the literature has emphasized that the implementation of multiple evidence-based elements can markedly improve perioperative outcomes.^37^ Several components of ERAS protocols, including early mobilization, standardized prophylactic antibiotics, and multimodal analgesic regimens, promote early ambulation and accelerate recovery, which ultimately prepare the patient for earlier discharge.^3^^,^^38^^,^^39^

Our meta-analysis showed a mean reduction in hospital LOS of -17.05 hours with ERAS compared with standard care. This reduction possibly reflects earlier discharge and improved patient experience,^3^^,^^40^ and aligns with previous meta-analyses reporting reductions of 12−14 hours, although they also showed similar high heterogeneity between included studies.^10^^,^^11^ Shorter hospital stays can improve hospital efficiency and resource optimization, and reduced LOS is widely used as a benchmark of institutional performance across diverse healthcare settings.^41^^,^^42^ Importantly, the reduction in hospital LOS was not associated with higher readmission rates, consistent with prior evidence.^8^^,^^10^^,^^11^ Nonetheless, discharge policies may vary across institutions, and we could not conclusively address resource utilization or cost-effectiveness due to limited reported data.

Pain management remains a critical factor in postoperative recovery, as it influences the quality of recovery and affects mother-newborn bonding in the postpartum period.^43^, ^44^, ^45^, ^46^, ^47^ Our pooled analysis suggests that ERAS may reduce pain at rest after cesarean delivery, with mean decreases of 1.32-point at 24 hours and 0.83-point at 48 hours, approaching the lower threshold of clinical significance. Although some studies suggest that the MCID for pain reduction on the VAS may be approximately 1.0-point (10 mm) on a 100 mm scale,^48^^,^^49^ in our analysis, we adopted a more conservative threshold of approximately 1.5-point based on evidence from gynecologic surgery. These findings are consistent with previous meta-analyses, in which the implementation of the ERAS protocol resulted in statistically significant reductions in postoperative pain and opioid consumption after cesarean delivery^8^; nonetheless, our results should be interpreted with caution, since this MCID value was extrapolated from hysterectomy studies due to a lack of a specific post-cesarean MCID in the current literature.

Wound infection after cesarean delivery is a major concern in clinical practice, as it is associated with increased maternal morbidity, delayed recovery, and hospital readmissions.^50^^,^^51^ In our meta-analysis, ERAS protocols were associated with a 77% relative reduction in wound infection risk. This finding is consistent with ERAS recommendations that emphasize evidence-based measures such as glycemic control in patients with diabetes, smoking cessation support, intravenous prophylactic antibiotics, chlorhexidine-alcohol skin preparation, prevention of intraoperative hypothermia, and early mobilization, all of which are directly related to lower surgical site infection rates.^5^

Maternal satisfaction is a multidimensional endpoint that captures the patient’s perception of care. It reflects the mother’s experience and influences postpartum mental health and quality of care.^52^, ^53^, ^54^, ^55^ Despite high variability among measurement tools, we found that maternal satisfaction was significantly higher among patients managed under ERAS protocols than among those under standard care. Beyond clinical recovery through multimodal analgesia and early mobilization, ERAS pathways may also improve communication, reduce anxiety, and strengthen trust between patients and clinicians.^56^^,^^57^ Together, these findings suggest that ERAS benefits may extend to both clinical and patient-centered outcomes; nonetheless, we had to pool different measurement tools into a common scale for synthesis, and the heterogeneity among scales may not adequately translate into comparable conclusions across studies.^23^

Emergency cesarean deliveries are associated with higher rates of maternal complications, longer recovery, and prolonged hospitalization.^58^ In our subgroup analysis by cesarean categories, ERAS protocols were associated with a significant reduction in hospital LOS for both elective and emergency procedures, with the largest effect observed in emergency cases (MD = -29.04 hours). This finding could be clinically relevant because emergency cesarean deliveries must meet tight decision-to-delivery intervals, limiting opportunities for preoperative optimization and structured perioperative preparation, which may contribute to longer recovery and increased hospital LOS in these higher-acuity cases.^59^ Our findings suggest that integrating ERAS pathways into perioperative care could possibly help streamline postoperative recovery and reduce unnecessary hospital stays in these higher-risk scenarios.

A larger reduction in length of stay was observed in LMIC (MD = -22.81 hours), whereas no significant effect was detected in UMIC. While this could reflect more established perioperative pathways and baseline efficiency in these settings, the test for subgroup differences did not achieve statistical significance, possibly due to the smaller number of included studies within the UMIC classification, which hinders any conclusive result. Nonetheless, in resource-constrained health systems, where surgical services are frequently limited by hospital overcrowding and a backlog of untreated surgical conditions, efficiency improvements that reduce hospital stay may enhance patient flow and access to surgical care.^1^ Albeit not directly addressed in our study, such improvements could have particular implications given that cesarean-related maternal mortality is nearly 100 times higher in LMIC than in high-income countries.^60^ Thus, the implementation of standardized ERAS pathways could possibly impact LMIC by improving efficiency and expanding safe surgical capacity, although further studies are necessary to draw solid evidence, given the lack of subgroup statistical significance in our analysis.

Still, the reliability and interpretability of our pooled estimates were substantially limited by the high heterogeneity observed across key outcomes, particularly hospital LOS (*I*^2^ = 98.2%), pain at 24 hours (*I*^2^ = 98.9%), pain at 48 hours (*I*^2^ = 95.2%), and maternal satisfaction (*I*^2^ = 94.9%). Accordingly, the GRADE certainty of the evidence was rated as very low for most outcomes given such heterogeneity. Our findings should therefore be interpreted as suggestive rather than definitive, since several factors likely contribute to this variability. As shown in Table 1, ERAS protocols differed considerably across trials in their composition, timing, and intensity within all three perioperative phases: preoperative fasting thresholds were heterogeneous, analgesic regimens varied from intrathecal morphine to TAP blocks and multimodal oral regimens, and postoperative mobilization targets differed in onset time. The definitions of “standard care” were similarly heterogeneous, reflecting local institutional practices rather than a uniform comparator. Discharge criteria and LOS measurement also varied, as some studies defined LOS as time to actual discharge, while others used fulfillment of institutional discharge criteria. Pain and satisfaction assessments also lacked standardization across studies, as previously discussed. Taken together, these sources of clinical and methodological heterogeneity represent an intrinsic limitation of this meta-analysis, in which data from diverse ERAS protocols were pooled for statistical purposes. Therefore, ERAS should be interpreted as a flexible framework of perioperative care rather than a single, standardized intervention. Accordingly, the effect estimates should be understood as averages across diverse implementations that may not be generalizable to any specific protocol or healthcare setting, although the overall direction of effect suggests benefit.

### Limitations

Our meta-analysis has several limitations. We observed considerable variation in the ERAS protocols across the included trials, which prevented the identification of a universally standardized obstetric ERAS pathway (Table 1). Similarly, “standard care” was inconsistently defined and largely dependent on local institutional practices, contributing to high heterogeneity and lowering the overall certainty of the evidence in the GRADE assessments. Incomplete reporting of perioperative elements in several RCTs limited the assessment of protocol adherence and its impact on key outcomes, including hospital LOS, which could be influenced by institutional discharge policies as well as by the ERAS protocol.

The definition of ‘emergency cesarean’ was not explicitly reported, limiting the interpretation of the clinical severity of included cases. Variability in the assessment of pain and maternal satisfaction, including differences in timing, scales, and interpretation, likely introduced measurement bias and reduced comparability across studies, contributing to the high heterogeneity observed for these outcomes. For example, the MCID was derived from studies of gynecologic surgery since there is no standard value for a post-cesarean MCID, and this may not fully reflect a clinically meaningful difference for parturients; the conversion of satisfaction measures into a single scale may also not fully reflect clinical similarities. Moreover, none of the trials reported data on cost-effectiveness, staff workload, or resource utilization, limiting our evaluation of ERAS as a potential system-strengthening intervention in resource-limited settings.

Finally, most included studies were judged as having some concerns regarding risk of bias, consistent with the GRADE assessment, which indicated low certainty of evidence for most outcomes, with only one outcome rated moderate. To evaluate the robustness of these findings, we conducted leave-one-out sensitivity analyses and TSA, which supported the stability of the results. However, these analyses do not reduce the clinical and methodological heterogeneity among included studies, and the findings should be interpreted considering the very low certainty of the evidence for most endpoints.

## Conclusion

This systematic review and meta-analysis provides updated, albeit still heterogeneous, evidence suggesting that ERAS protocols may provide additional benefits for women undergoing cesarean delivery, including shorter hospital LOS, lower postoperative pain scores at 24 and 48 hours, reduced wound infection rates, and higher maternal satisfaction, although conclusions across different healthcare settings were limited. Importantly, ERAS represents a flexible framework that can be adapted to local needs and clinical contexts. Future research should focus not only on demonstrating efficacy but also on achieving sustainable implementation through multidisciplinary education, local adaptation, and cultural integration within diverse health systems.

## Data availability statement

This study was based on data extracted from previously published trials. The authors do not have access to patient-level data from individual publications. All data supporting the findings of this study are available within the article and its Supplementary Material. On July 27, 2026, all included trials and key cited references were checked for retractions, corrections, withdrawals, and expressions of concern in PubMed and on the corresponding journal or publisher websites. No included study or key reference was found to have been retracted or flagged.

## Authors’ contributions

Tauãna Terra: Conceptualization, Methodology, Investigation, Visualization, Writing-original draft, Writing-review & editing. Vanessa Tapioca: Conceptualization, Investigation, Writing-original draft, Writing-review & editing. Eduardo Pereira: Conceptualization, Formal analysis, Visualization, Writing-original draft, Writing-review & editing. Bruna Ferreira: Investigation, Visualization. Tamiris Soares: Investigation, Visualization, Writing-original draft. Sara Amaral: Conceptualization, Methodology. Maria Luisa Assis: Investigation, Visualization. Gabriel Carvalho: Investigation, Writing-review & editing. Julio S. Curi: Investigation. Kamal Kumar: Writing-original draft, Writing-review & editing, Supervision. Agreement to be accountable for all aspects of the work, thereby ensuring that questions related to the accuracy and integrity of any part of the work are appropriately investigated and resolved: all authors.

All authors (1) read and approved the final version, (2) met the ICMJE criteria for authorship, (3) believe the paper represents honest work, and (4) are able to verify the validity of the results reported.

## Declaration of Artificial Intelligence (AI) use

The authors did not use AI or AI-assisted technologies at any stage of this work, including data extraction, statistical analysis, reference checking, figure preparation, preparation of the supplementary material, and language editing.

## Funding

This research did not receive any specific grant from funding agencies in the public, commercial, or not-for-profit sectors.

## PROSPERO registry

CRD420251152114 (October 23, 2025).

## Disclosures

This manuscript is an original work; it has not been presented as a conference abstract or submitted to other journals.

## Conflicts of interest

The authors declare no have conflicts of interest.

## Appendix Supplementary materials

Image, application 1

## Footnotes

Supplementary material associated with this article can be found in the online version at doi:10.1016/j.bjane.2026.844817.
