# Supplementary material for: Enhanced recovery after surgery for cesarean delivery: a systematic review and meta-analysis of randomized trials with trial sequential analysis
Source: Braz J Anesthesiol. 2026 Aug 14;76(5):844817. doi: 10.1016/j.bjane.2026.844817 (PMC13572303; doi:10.1016/j.bjane.2026.844817)
Supplement: Supplementary file 1 [file mmc1.docx]

**BJAN-D-26-00155**

**Supplementary Material For:**

**Enhanced recovery after surgery for cesarean delivery: a systematic review and meta-analysis of randomized trials with trial sequential analysis**

1. Supplementary Table S1 ……………………………………………………. Complete search strategy
2. Supplementary Table S2 ……………………………………………………. Outcome definitions for individual studies
3. Supplementary Table S3 ……………………………………………………. List of excluded studies and reasons
4. Supplementary Figure S1 ………………………………………………. Risk of bias assessment for individual studies
5. Supplementary Figure S2 ………………………………………………. Leave-one-out sensitivity analyses
6. Supplementary Table S4 ……………………….…………………………. GRADE summary of findings table
7. Supplementary Figure S3 ………………………………… Hospital length of stay by cesarean type (elective, emergency, or mixed subgroups)
8. Supplementary Figure S4 …………………………………. Wound infection
9. Supplementary Figure S5 …………………………………. Readmission
10. Supplementary Figure S6 …………………………………. Postoperative nausea and vomiting
11. Supplementary Figure S7 …………………………………. Maternal satisfaction score
12. Supplementary Table S5 …………………………………. Data extraction for all meta-analyzed outcomes
13. Supplementary File S1 ….…………………………………. R and TSA code used to reproduce the analyses

1. **Supplementary Table S1**. Complete search strategy.

| **Cochrane search strategy:** | ("ERAS" OR "Enhanced Recovery after Surgery" OR "ERAC" OR "Enhanced Recovery after Cesarean") AND (cesarean OR "c-section" OR caesarean) |
| --- | --- |
| **Embase search strategy:** | ('eras' OR 'enhanced recovery after surgery' OR 'enhanced recovery after surgery'/exp OR 'erac' OR 'enhanced recovery after cesarean') AND ('cesarean' OR 'cesarean section'/exp OR 'c-section') |
| **PubMed search strategy:** | ("ERAS" OR "Enhanced Recovery after Surgery" OR "Enhanced Recovery After Surgery"[MeSH Terms] OR "ERAC" OR "Enhanced Recovery after Cesarean") AND (cesarean OR "Cesarean Section"[MeSH Terms] OR "c-section") |
| **Web of Science search strategy:** | TS=(ERAS OR "Enhanced Recovery after Surgery" OR ERAC OR "Enhanced Recovery after Cesarean") AND TS=(cesarean OR caesarean OR "cesarean section" OR "c-section") |

2. **Supplementary Table S2**. Outcome definitions for individual studies.

| **Author,**  **year** | **Type of cesarean** | **Hospital LOS** | **Pain score 24h postoperatively** | **Pain score 48h postoperatively** | **Wound infection** | **Readmission** | **PONV** | **Maternal satisfaction score** |
| --- | --- | --- | --- | --- | --- | --- | --- | --- |
| Abilashini, 2025 | Emergency | Total length of stay | Pain score at rest ^a^ | NA | NA | NA | NA | Maternal satisfaction^b^ |
| Baluku, 2020 | Emergency | Time of surgery to discharge | NA | NA | Wound infection up to 30 days postoperatively | Readmissions up to 30 days postoperatively | PONV within the first 24 hours | NA |
| Birden, 2025 | Elective | NA | VAS score at rest | NA | Surgical site infection | Readmissions up to 30 days postoperatively | PONV | NA |
| Darwish, 2022 | Elective | NA | NA | NA | Wound infection | NA | NA | NA |
| Emelinda, 2024 | Elective | NA | NA | NA | NA | Hospital readmission | PONV | NA |
| Kanabar, 2024 | Elective | Time from the start of the operation to discharge | NA | NA | NA | NA | NA | NA |
| Klangprapan, 2022 | Elective | NA | VAS at rest | NA | NA | NA | NA | NA |
| Kulshrestha, 2025 | Elective | NA | NRS at rest | NRS at rest | NA | NA | NA | Likert scale of five in postoperative period |
| Mundhra, 2024 | Emergency | Time to patient being fit for discharge (ambulating, tolerating a normal diet, urinating, and experiencing tolerable pain with oral medications) | VAS at rest | VAS at rest | NA | Hospital readmission within 30 days of discharge | NA | NA |
| Pan, 2020 | Elective | Total length of stay | VAS at rest | VAS at rest | NA | NA | Postoperative nausea | Satisfaction VAS |
| Rajendran, 2025 | Elective | Total length of stay | VAS at rest | VAS at rest | NA | NA | Postoperative nausea | Satisfaction VAS |
| Teigen, 2020 | Elective | Hours | NA | NA | Wound infection | Hospital readmission | NA | NA |
| Xu, 2025 | Mixed | Time of surgery to discharge | VAS at rest | VAS at rest | NA | NA | NA | PSNCQQ |

^a^Measurement method for pain intensity was not specified.

^b^Measurement instrument for maternal satisfaction was not mentioned.

Hospital LOS: hospital length of hospital stay.

NRS: numerical rating scale.

PONV: postoperative nausea and vomiting.

PSNCQQ: Patient Satisfaction with Nursing Care Quality Questionnaire.

Satisfaction VAS is a 10-cm scale used to measure their satisfaction level, where a score of 0 represents “extremely unsatisfied” and 10 indicates extremely satisfied.

VAS: visual analog scale.

3. **Supplementary Table S3.** List of excluded studies and reasons.

| **Author, year** | **DOI, PMID, reference, or link** | **Reason for exclusion** |
| --- | --- | --- |
| Easa, 2024 | DOI 10.1093/qjmed/hcae175.579 | Abstract only |
| Cai, 2024 | DOI 10.29063/ajrh2024/v28i12.17 | Wrong intervention |
| Mohamed, 2025 | DOI 10.21608/mjmr.2025.403486.2027 | No access to the article |
| Nittiwatthanawit, 2025 | DOI 10.31083/CEOG42810 | Wrong intervention |
| Beedkar, 2025 | DOI 10.7860/JCDR/2025/81157.21595 | No outcomes of interest |

DOI, Digital Object Identifier; PMID, PubMed Identifier.

4. **Supplementary Figure S1.** Risk of bias assessment for individual studies.

5. **Supplementary Figure S2.** Leave-one-out sensitivity analyses.

Hospital length of stay

Pain scores at rest at 24 hours

Pain scores at rest at 48 hours

Wound infection

Readmission

Postoperative nausea and vomiting

Maternal satisfaction score

6. **Supplementary Table S4.** GRADE summary of findings table.

| **Outcomes** | **№ of participants (studies) Follow-up** | **Certainty of the evidence (GRADE)** | **Relative effect (95% CI)** | **Anticipated absolute effects** | |
| --- | --- | --- | --- | --- | --- |
|  |  |  |  | **Risk with Standard** | **Risk difference with ERAS** |
| LOS | 1128 (8 RCTs) | ⨁◯◯◯ Very low^a,b^ | - | The mean LOS ranged from **-58.80 to 1.44** hours | MD **17.05 hours lower** (25.08 lower to 9.02 lower) |
| Pain score 24h | 1097 (8 RCTs) | ⨁◯◯◯ Very low^a,b,c^ | - | The mean Pain 24h ranged from **-2.61 to -0.29** points | MD **1.32 points lower** (2.27 lower to 0.37 lower) |
| Pain score 48h | 798 (5 RCTs) | ⨁◯◯◯ Very low^a,b,c^ | - | The mean Pain 48h ranged from **-1.87 to -0.24** points | MD **0.83 points lower** (1.56 lower to 0.09 lower) |
| Wound infection | 727 (4 RCTs) | ⨁⨁⨁◯ Moderate^d^ | **RR 0.23** (0.07 to 0.73) | 44 per 1.000 | **34 fewer per 1.000** (41 fewer to 12 fewer) |
| Readmission | 611 (5 RCTs) | ⨁◯◯◯ Very low^a,c,d^ | **RR 0.59** (0.23 to 1.55) | 72 per 1.000 | **29 fewer per 1.000** (55 fewer to 39 more) |
| PONV | 667 (5 RCTs) | ⨁◯◯◯ Very low^a,c,d^ | **RR 0.65** (0.36 to 1.19) | 79 per 1.000 | **28 fewer per 1.000** (50 fewer to 15 more) |
| Maternal satisfaction score | 756 (5 RCTs) | ⨁◯◯◯ Very low^a,b,d^ | - | - | SMD **1.02 SD higher** (0.33 higher to 1.72 higher) |
| ***The risk in the intervention group** (and its 95% confidence interval) is based on the assumed risk in the comparison group and the **relative effect** of the intervention (and its 95% CI).  **CI:** confidence interval; **MD:** mean difference; **RR:** risk ratio; **SMD:** standardized mean difference. | | | | | |
| **GRADE Working Group grades of evidence** **High certainty:** we are very confident that the true effect lies close to that of the estimate of the effect. **Moderate certainty:** we are moderately confident in the effect estimate: the true effect is likely to be close to the estimate of the effect, but there is a possibility that it is substantially different. **Low certainty:** our confidence in the effect estimate is limited: the true effect may be substantially different from the estimate of the effect. **Very low certainty:** we have very little confidence in the effect estimate: the true effect is likely to be substantially different from the estimate of effect. | | | | | |

**Explanations**

a. The outcome was influenced by studies with some concerns for risk of bias, warranting a one-level downgrade in evidence certainty.

b. The outcome exhibited substantial heterogeneity. Downgraded by two levels due to inconsistency.

c. The outcome exhibited wide confidence intervals. Downgraded by one level due to imprecision.

d. Few studies reported this outcome. Downgraded by one level due to publication bias.

7. **Supplementary Figure S3.** Forest plot depicting the endpoint of hospital length of stay, stratified by cesarean type. ERAS, Enhanced Recovery After Surgery; SD, standard deviation; CI, confidence interval; MD, mean difference.

8. **Supplementary Figure S4.** Forest plot depicting the endpoint of wound infection. ERAS, Enhanced Recovery After Surgery; CI, confidence interval; RR, risk ratio.

9. **Supplementary Figure S5.** Forest plot depicting the endpoint of readmission. ERAS, Enhanced Recovery After Surgery; CI, confidence interval; RR, risk ratio.

10. **Supplementary Figure S6.** Forest plot depicting the endpoint of postoperative nausea and vomiting. ERAS, Enhanced Recovery After Surgery; CI, confidence interval; RR, risk ratio.

11. **Supplementary Figure S7.** Forest plot depicting the endpoint of maternal satisfaction score. ERAS, Enhanced Recovery After Surgery; SD, standard deviation; CI, confidence interval; SMD, standardized mean difference.

12. **Supplementary Table S5**. Data extraction for all meta-analyzed outcomes.

Hospital length of stay - subgroup analysis by type of cesarean

| **Author** | **Subgroup** | **mean.e** | **sd.e** | **n.e** | **mean.c** | **sd.c** | **n.c** |
| --- | --- | --- | --- | --- | --- | --- | --- |
| Abilashini, 2025 | Emergency | 76.80 | 26.40 | 50 | 129.60 | 31.20 | 50 |
| Baluku, 2020 | Emergency | 43.60 | 13.70 | 80 | 62.10 | 17.30 | 80 |
| Mundhra, 2024 | Emergency | 53.87 | 15.02 | 71 | 73.92 | 8.96 | 71 |
| Kanabar, 2024 | Elective | 63.93 | 6.45 | 46 | 69.80 | 2.81 | 46 |
| Pan, 2020 | Elective | 112.08 | 11.76 | 112 | 110.64 | 15.36 | 104 |
| Rajendran, 2025 | Elective | 90.24 | 17.45 | 50 | 112.32 | 17.10 | 50 |
| Teigen, 2020 | Elective | 73.77 | 4.21 | 58 | 75.04 | 3.02 | 60 |
| Xu, 2025 | Mixed | 102.40 | 9.94 | 100 | 127.20 | 10.30 | 100 |

Hospital length of stay - subgroup analysis by country income level

| **Author** | **Subgroup** | **mean.e** | **sd.e** | **n.e** | **mean.c** | **sd.c** | **n.c** |
| --- | --- | --- | --- | --- | --- | --- | --- |
| Abilashini, 2025 | LMIC | 76.80 | 26.40 | 50 | 129.60 | 31.20 | 50 |
| Baluku, 2020 | LMIC | 43.60 | 13.70 | 80 | 62.10 | 17.30 | 80 |
| Kanabar, 2024 | LMIC | 63.93 | 6.45 | 46 | 69.80 | 2.81 | 46 |
| Mundhra, 2024 | LMIC | 53.87 | 15.02 | 71 | 73.92 | 8.96 | 71 |
| Rajendran, 2025 | LMIC | 90.24 | 17.45 | 50 | 112.32 | 17.10 | 50 |
| Pan, 2020 | UMIC | 112.08 | 11.76 | 112 | 110.64 | 15.36 | 104 |
| Teigen, 2020 | UMIC | 73.77 | 4.21 | 58 | 75.04 | 3.02 | 60 |
| Xu, 2025 | UMIC | 102.40 | 9.94 | 100 | 127.20 | 10.30 | 100 |

Pain score at rest at 24 hours (0–10 scale)

| **Author** | **mean.e** | **sd.e** | **n.e** | **mean.c** | **sd.c** | **n.c** |
| --- | --- | --- | --- | --- | --- | --- |
| Abilashini, 2025 | 2.20 | 0.70 | 50 | 3.90 | 1.20 | 50 |
| Birden, 2025 | 4.70 | 7.55 | 78 | 5.29 | 7.55 | 78 |
| Klangprapan, 2022 | 3.10 | 1.90 | 22 | 5.10 | 1.90 | 21 |
| Kulshrestha, 2025 | 2.13 | 0.11 | 70 | 4.74 | 0.11 | 70 |
| Mundhra, 2024 | 8.08 | 1.09 | 71 | 8.96 | 1.10 | 71 |
| Pan, 2020 | 2.46 | 1.58 | 112 | 3.50 | 1.76 | 104 |
| Rajendran, 2025 | 1.76 | 0.82 | 50 | 2.96 | 0.95 | 50 |
| Xu, 2025 | 7.35 | 0.75 | 100 | 7.64 | 0.75 | 100 |

Pain score at rest at 48 hours (0–10 scale)

| **Author** | **mean.e** | **sd.e** | **n.e** | **mean.c** | **sd.c** | **n.c** |
| --- | --- | --- | --- | --- | --- | --- |
| Kulshrestha, 2025 | 1.61 | 0.09 | 70 | 3.48 | 0.90 | 70 |
| Mundhra, 2024 | 6.14 | 0.85 | 71 | 6.87 | 1.50 | 71 |
| Pan, 2020 | 2.00 | 1.65 | 112 | 2.60 | 1.57 | 104 |
| Rajendran, 2025 | 2.30 | 0.86 | 50 | 2.54 | 0.93 | 50 |
| Xu, 2025 | 6.35 | 2.25 | 100 | 7.00 | 1.50 | 100 |

Wound infection (number of events)

| **Author** | **event.e** | **n.e** | **event.c** | **n.c** |
| --- | --- | --- | --- | --- |
| Baluku, 2020 | 2 | 76 | 5 | 77 |
| Birden, 2025 | 0 | 78 | 3 | 78 |
| Darwish, 2022 | 0 | 150 | 4 | 150 |
| Teigen, 2020 | 0 | 58 | 4 | 60 |

Readmission (number of events)

| **Author** | **event.e** | **n.e** | **event.c** | **n.c** |
| --- | --- | --- | --- | --- |
| Baluku, 2020 | 0 | 76 | 2 | 77 |
| Birden, 2025 | 11 | 78 | 15 | 78 |
| Emelinda, 2024 | 0 | 21 | 0 | 21 |
| Mundhra, 2024 | 1 | 71 | 0 | 71 |
| Teigen, 2020 | 0 | 58 | 5 | 60 |

Postoperative nausea and vomiting (number of events)

| **Author** | **event.e** | **n.e** | **event.c** | **n.c** |
| --- | --- | --- | --- | --- |
| Baluku, 2020 | 5 | 76 | 6 | 77 |
| Birden, 2025 | 2 | 78 | 5 | 78 |
| Emelinda, 2024 | 4 | 21 | 7 | 21 |
| Pan, 2020 | 4 | 112 | 1 | 104 |
| Rajendran, 2025 | 3 | 50 | 7 | 50 |

Maternal satisfaction score (0–10 scale)

| **Author** | **mean.e** | **sd.e** | **n.e** | **mean.c** | **sd.c** | **n.c** |
| --- | --- | --- | --- | --- | --- | --- |
| Abilashini, 2025 | 8.50 | 1.20 | 50 | 6.20 | 1.70 | 50 |
| Kulshrestha, 2025 | 8.80 | 1.20 | 70 | 6.80 | 1.56 | 70 |
| Pan, 2020 | 8.42 | 0.76 | 112 | 8.12 | 8.20 | 104 |
| Rajendran, 2025 | 6.18 | 0.90 | 50 | 4.76 | 0.62 | 50 |
| Xu, 2025 | 9.34 | 1.54 | 100 | 8.82 | 1.39 | 100 |

Column labels follow the notation of the meta R package: the suffix .e refers to the ERAS group and the suffix .c to the standard care group. mean: mean value; sd: standard deviation; n: number of participants analyzed; event: number of participants with the event. ERAS: Enhanced Recovery After Surgery; LMIC: low- and middle-income countries; UMIC: upper-middle-income countries. The pooled estimate for hospital length of stay is identical in both subgroup analyses, which differ only in the stratifying variable. Hospital length of stay is expressed in hours. Pain and maternal satisfaction scores were rescaled to a common 0–10 scale, with higher values indicating more intense pain and greater satisfaction, respectively.

13. **Supplementary File S1**. R and TSA code used to reproduce the analyses.
- *R Codes*

**# INSTALL PACKAGES**

**install.packages("meta")**

**install.packages("metafor")**

**install.packages("readr")**

**install.packages("readxl")**

**# LOAD PACKAGES**

**library(meta)**

**library(metafor)**

**library(readr)**

**library(readxl)**

**library(readr)**

**# CONTINUOUS OUTCOMES - CAPTIONS TO NAME EACH TABLE FILE IN .CSV**

**Hospital Length of Stay = HLOS**

**Low- and middle-income country subgroup = LMIC**

**Upper-middle-income country subgroup = UMIC**

**Elective cesarean subgroup = Elective**

**Emergency cesarean subgroup = Emergency**

**Pain Score 24h Rest = PS24R**

**Pain Score 48h Rest = PS48R**

**Maternal Satisfaction Score = MSS**

**# BINARY OUTCOMES - CAPTIONS TO NAME EACH TABLE FILE IN .CSV**

**Wound Infection = WI**

**Readmission = READM**

**PONV = PONV**

**##### CONTINUOUS OUTCOMES #####**

**# 1 RUN META-ANALYSIS**

**library(readr)**

**TABLE_CAPTION <- read_delim("C:/Users/TABLE_CAPTION.csv", # CHANGE PATHWAY TO FILE + REPLACE “TABLE_CAPTION” FOR THE TABLE CAPTION**

**delim = ";", escape_double = FALSE, trim_ws = TRUE)**

**m.TABLE_CAPTION<-metacont(n.e=n.e, mean.e=mean.e, sd.e=sd.e, n.c=n.c, mean.c=mean.c, sd.c=sd.c, # REPLACE “TABLE_CAPTION” in “m.TABLE_CAPTION” FOR THE TABLE CAPTION**

**data = TABLE_CAPTION, # REPLACE “TABLE_CAPTION” FOR THE TABLE CAPTION**

**method.tau = "DL",**

**sm = "MD", # REPLACE TO SMD WHEN ASSESSING MSS**

**hakn = FALSE,**

**studlab = Author)**

**# 2 RUN LEAVE-ONE-OUT ANALYSES**

**Leaveoneout_m.TABLE_CAPTION<-metainf(m.TABLE_CAPTION, # REPLACE “TABLE_CAPTION” in “m.TABLE_CAPTION” FOR THE TABLE CAPTION**

**pooled="random")**

**summary(Leaveoneout_m.TABLE_CAPTION) # REPLACE “TABLE_CAPTION” in “m.TABLE_CAPTION” FOR THE TABLE CAPTION**

**forest(Leaveoneout_m.TABLE_CAPTION, # REPLACE “TABLE_CAPTION” in “m.TABLE_CAPTION” FOR THE TABLE CAPTION**

**col.square="lightblue",**

**col.diamond="gray",**

**xlim=c(-20, 20), # ADJUST AS NEEDED FOR FORMATTING PURPOSES**

**xlab="Favors TABLE_CAPTION Favors TABLE_CAPTION", # REPLACE “TABLE_CAPTION” FOR EACH GROUP DEPENDING ON THE DIRECTION OF EFFECT AND THE CLINICAL REASON**

**ff.xlab = "bold",**

**rightcols = c("effect", "ci", "I2"))**

**# RUN FUNNEL PLOT**

**funnel(m.TABLE_CAPTION, # REPLACE “TABLE_CAPTION” in “m.TABLE_CAPTION” FOR THE TABLE CAPTION**

**xlab = "MD", # REPLACE TO SMD WHEN ASSESSING MSS**

**studlab = TRUE,**

**cex.studlab = 0.6,**

**xlim = c(-15, 15), # ADJUST AS NEEDED FOR FORMATTING PURPOSES**

**contour = c(0.90, 0.95, 0.99),**

**col.contour = c("white", "white", "white"),**

**legend.pos = "topright")**

**# 4 RUN FOREST PLOT**

**forest(m.TABLE_CAPTION, # REPLACE “TABLE_CAPTION” in “m.TABLE_CAPTION” FOR THE TABLE CAPTION**

**layout="Revman",**

**sortvar=studlab,**

**leftcols=c("studlab","mean.e","sd.e","n.e",**

**"mean.c","sd.c","n.c","w.random", "effect", "ci"),**

**leftlabs = c("Studies","Mean","SD", "Total",**

**"Mean","SD","Total","Weight","MD","95% CI"), # REPLACE TO SMD WHEN ASSESSING MSS AND CHANGE UNIT LABEL (MEAN, SD, MD) AS PER OUTCOME**

**rightcols=FALSE,**

**just.addcols="right",**

**random=TRUE,**

**common=FALSE,**

**pooled.events=TRUE,**

**pooled.totals = TRUE,**

**test.overall.random=TRUE,**

**overall.hetstat=TRUE,**

**digits=2,**

**digits.pval=2,**

**digits.sd = 2,**

**col.square="darkblue", col.square.lines="black",**

**col.diamond="black", col.diamond.lines="black",**

**diamond.random=TRUE,**

**lab.e="TABLE_CAPTION", # REPLACE “TABLE_CAPTION” FOR EACH GROUP DEPENDING ON THE DIRECTION OF EFFECT AND THE CLINICAL REASON**

**lab.c="TABLE_CAPTION", # REPLACE “TABLE_CAPTION” FOR EACH GROUP DEPENDING ON THE DIRECTION OF EFFECT AND THE CLINICAL REASON**

**fs.heading=12,**

**colgap.forest = "6mm", # ADJUST AS NEEDED FOR FORMATTING PURPOSES**

**label.left="Favors TABLE_CAPTION", col.label.left = "black", ff.lr = "bold", # REPLACE “TABLE_CAPTION” FOR EACH GROUP DEPENDING ON THE DIRECTION OF EFFECT AND THE CLINICAL REASON**

**label.right="Favors TABLE_CAPTION", col.label.right = "black", # REPLACE “TABLE_CAPTION” FOR EACH GROUP DEPENDING ON THE DIRECTION OF EFFECT AND THE CLINICAL REASON**

**digits.tau2 = 2,**

**print.pval.Q = TRUE, print.tau.ci = TRUE)**

**##### BINARY OUTCOMES #####**

**# 1 RUN META-ANALYSIS**

**library(readr)**

**TABLE_CAPTION <- read_delim("C:/Users/TABLE_CAPTION.csv", # CHANGE PATHWAY TO FILE AND REPLACE “TABLE_CAPTION” FOR THE TABLE CAPTION**

**delim = ";", escape_double = FALSE, trim_ws = TRUE)**

**m.TABLE_CAPTION<-metabin (event.e, n.e, event.c, n.c, # REPLACE “TABLE_CAPTION” in “m.TABLE_CAPTION” FOR THE TABLE CAPTION**

**data = TABLE_CAPTION, # REPLACE “TABLE_CAPTION” FOR THE TABLE CAPTION**

**method = "MH",**

**method.tau = "DL",**

**sm = "RR",**

**hakn = FALSE,**

**studlab = Author)**

**# 2 RUN LEAVE-ONE-OUT ANALYSES**

**TABLE_CAPTION <- read_delim("C:/Users/TABLE_CAPTION.csv", # CHANGE PATHWAY TO FILE + REPLACE “TABLE_CAPTION” FOR THE TABLE CAPTION**

**delim = ";", escape_double = FALSE, trim_ws = TRUE)**

**summary(Leaveoneout_m.TABLE_CAPTION) # REPLACE “TABLE_CAPTION” in “m.TABLE_CAPTION” FOR THE TABLE CAPTION**

**forest(Leaveoneout_m.TABLE_CAPTION, # REPLACE “TABLE_CAPTION” in “m.TABLE_CAPTION” FOR THE TABLE CAPTION**

**col.square="lightblue",**

**col.diamond="gray",**

**xlim=c(0.2, 2), # ADJUST AS NEEDED FOR FORMATTING PURPOSES**

**xlab="Favors TABLE_CAPTION Favors TABLE_CAPTION", # REPLACE “TABLE_CAPTION” FOR EACH GROUP DEPENDING ON THE DIRECTION OF EFFECT AND THE CLINICAL REASON**

**ff.xlab = "bold",**

**rightcols = c("effect", "ci", "I2"))**

**# 3 RUN FUNNEL PLOTS**

**funnel(m.TABLE_CAPTION, # REPLACE “TABLE_CAPTION” in “m.TABLE_CAPTION” FOR THE TABLE CAPTION**

**xlab = "RR",**

**studlab = TRUE,**

**cex.studlab = 0.6,**

**xlim = c(0.1, 5), # ADJUST AS NEEDED FOR FORMATTING PURPOSES**

**contour = c(0.90, 0.95, 0.99),**

**col.contour = c("white", "white", "white"),**

**legend.pos = "topright")**

**# 4 RUN FOREST PLOTS**

**forest(m.TABLE_CAPTION, # REPLACE “TABLE_CAPTION” in “m.TABLE_CAPTION” FOR THE TABLE CAPTION**

**layout="Revman",**

**sortvar=studlab,**

**lab.e="TABLE_CAPTION", lab.c="TABLE_CAPTION", # REPLACE “TABLE_CAPTION” FOR EACH GROUP DEPENDING ON THE DIRECTION OF EFFECT AND THE CLINICAL REASON**

**label.left="Favors TABLE_CAPTION", col.label.left = "black", ff.lr = "bold", # REPLACE “TABLE_CAPTION” FOR EACH GROUP DEPENDING ON THE DIRECTION OF EFFECT AND THE CLINICAL REASON**

**label.right="Favors TABLE_CAPTION", col.label.right = "black", # REPLACE “TABLE_CAPTION” FOR EACH GROUP DEPENDING ON THE DIRECTION OF EFFECT AND THE CLINICAL REASON**

**leftcols=c("studlab", "event.e", "n.e", "event.c", "n.c", "w.random", "effect", "ci"),**

**rightcols=FALSE,**

**pooled.events=TRUE,**

**pooled.totals=TRUE,**

**random=TRUE,**

**common=FALSE,**

**diamond.random=TRUE,**

**fs.heading=12,**

**fs.study = 12,**

**fs.hetstat = 12,**

**colgap="6mm", # ADJUST AS NEEDED FOR FORMATTING PURPOSES**

**digits=2,**

**digits.pval=2,**

**col.square="#darkblue", col.square.lines="black",**

**col.diamond="black", col.diamond.lines="black",**

**print.Q = TRUE, print.pval.Q = TRUE, print.tau.ci = TRUE,**

**test.overall.random=TRUE,**

**digits.tau2 = 2,**

**overall.hetstat=TRUE)**

**# FOR SUBGROUPS (CONTINUOUS OR BINARY)**

**“RUN META-ANALYSIS” SECTION: Add the argument “byvar = Subgroup” to the metacont() (continuous) or metabin() (binary) function call.**

**“RUN FOREST PLOT” SECTION: Add the arguments “bylab”, “col.by”, “fs.by”, “sep.by”, “colgap.by”, “print.bylabels”, “print.byvar”, and “print.subgroup.name” to the forest() function call**

- *TSA Code*

**Software:** TSA, version 0.9.21 (Copenhagen Trial Unit)

**Data source:** Import the study-level data (n, mean, SD) for HLOS from the table.

**Create new analysis** → Outcome type: Continuous → Model: DerSimonian-Laird random-effects.
**Enter trial data**: input n.e, mean.e, sd.e, n.c, mean.c, sd.c for each of the 8 included trials. Effect measure: mean difference (MD).

Alternatively, for HLOS’ TSA, the MD and 95% CIs can be recreated in Review Manager (RM) using the data extracted in the HLOS tables.

**This was done twice to cross-check any discrepancies between the two methods.**

The same statistical parameters from the original manuscript that were used in R were also used in the TSA software (and in RM for cross-check).
**Specify error thresholds**: Two-sided test; Type I error (alpha): 0.05; Power: 80% (Type II error, beta = 0.20); Alpha-spending function: O'Brien-Fleming.

**Set required information size (RIS) parameters**: Anticipated intervention effect, derived from the conventional random-effects results.

**Run the analysis** to generate the cumulative Z-curve, trial sequential monitoring boundaries, and RIS.
